# Contested skills and constrained mobilities: migrant carework skill regimes in Taiwan and Japan

**DOI:** 10.1186/s40878-022-00311-2

**Published:** 2022-09-13

**Authors:** Pei-Chia Lan

**Affiliations:** Department of Sociology, National Taiwan University, 1, Roosevelt Rd. Sec 4, Taipei, 106 Taiwan

**Keywords:** Migrant care worker, Labor migration, Guest worker, Skill regime, Japan, Taiwan

## Abstract

This article compares the paradoxical conditions of migrant care workers in two major receiving countries in Asia: Taiwan’s policy regime has positioned live-in care workers as “unskilled” foreigners, who nevertheless have gained increasing desirability and mobility in the labor market. By contrast, Japan has maintained the regime of skilled migration but the recent expansion of the trainee program reinforces paternalistic control over migrant caregivers, who are considered culturally inadequate. Contesting the assumption that skills indicate desirability and mobility in the labor market, I argue that we must examine the context-dependent constitution of skills at the intersection of migration, care, and skill regimes. I propose a multifaced framework to examine how the state and intermediary agencies co-produce the skill regime of care migration, including the following dimensions: migrant skills as a political language and structure of governance, care work skills as social and cultural constructions, the infrastructure of recruitment and training, and the consequence of labor market mobility.

## Introduction

Scholars have debated the significance of skill in the process of migrant selection and mobility. It is often assumed that skills can be objectively measured and converted into labor market rewards and opportunities. And yet, the skilled/unskilled binary is also a dubious boundary subject to cultural variations and political intervention. The nature of carework skills is especially contested because it is associated with women’s roles in the private sphere and largely staffed by minorities and immigrants. Scholars have accumulated rich studies about the migration–care nexus, but we still have limited knowledge about the formation of migrant carework skill regimes across cultural and policy contexts.

Compares two major receiving countries in Asia, Taiwan and Japan, this article offers a multifaced analytical framework for migrant carework skill regimes and identifies the innate contradictions and unsustainability across local particularities. Taiwan has positioned live-in care workers as “unskilled” foreigners who thereby lack access to long-term residency, but their labor is so desirable that they have gained increasing labor market mobility. By contrast, Japan has subscribed to the principle of skilled migration and yet the recent expansion of the trainee program (TITP) reinforces paternalistic control over migrant caregivers who are considered culturally inadequate.

The article is organized into five parts. First, I review the literature on the migration-care nexus and skill regime and propose my analytical framework and research questions. Second, I explain why it matters to compare Taiwan and Japan, and summarize my research methods. The third and fourth sections investigate the two countries separately, starting with an overview of relevant policies, followed by a discussion on state governance in alliance with intermediary agents, and ending with an assessment of the impact on migrant workers’ mobilities. Finally, the conclusion addresses the theoretical and policy implications of this research.

### Migrant carework skill regime

Scholars have used the intersection between *care regime* and *migration regime* to explain various policy patterns in the recruitment of migrant care workers. The former organizes long-term care between the state, market, family, and the voluntary sector, in association with particular cultural scripts about ideal care. The latter encompasses immigration policies and citizenship requirements that regulate the entry and status of foreigners (Da Roit & Weicht, 2013; Ogawa, 2018; Williams, 2012).

More recently, scholars have begun to investigate the formation of *skill regimes* in the recruitment of migrant workers. To examine skills as an institutional regime moves away from the neutral measurement of individual abilities or human capital, such as educational backgrounds, professional certificates, and language proficiency. Shan and Fejes (2015) characterize a skill regime as “a new mode of control and modulation that defines the *desirability* of individuals in the labor market” (my emphasis); such skill discourses not only cater to the productive interests of state and capital but are also closely linked to the social hierarchies of gender, race, and class, producing differential opportunities and unequal experiences for migrants.

The measurement of care work skills is even more contested, for several reasons. First, care work is highly associated with women’s family roles; when outsourced to the market, it is still feminized and often not considered “real work” or work that requires “professional skills.” Second, care work is further undervalued and underpaid when it is staffed by racial minorities and im/migrants, often because ethnic and cultural differences are suspected of being a barrier to the provision of high-quality care. Finally, care work is intimate labor and is often conducted in private households; the emotional and affective dimension of this labor is essential but ill-recognized; and care workers tend to have limited access to organizational support and legal protection.

For migrant workers who are classified by the host state as “unskilled,” their creativity and efforts are overlooked and their rights and personhood are deprived. Iskander (2021), in her ethnography of migrant construction workers in Qatar, explores the political and economic consequences of skill as a *language of power*. The political discourse that perceives skill as measurable human capital detached from embodied labor power justifies the marginal status and precarious work of lower-educated migrants. Hagan et al. (2015), in their study of Mexican migrants, also question the assumption of “unskilled” workers as a homogenous group who neither possess measurable competencies nor learn new ones, and face limited prospects of mobility in their migrant careers. Instead, they emphasize that migrants acquire valuable tacit skills through migration experience and routine tasks, and they can transfer them across regional and international markets to broaden their opportunities abroad and upon return.

Ortiga et al. (2021: p. 437) further argue that a “skills regime is not simply a discursive strategy for constructing ‘desirable’ workers, but a rationalized system of procedures and measures meant to evaluate one’s capabilities,” such as certification and training. By comparing the skilling programs catering to citizen and migrant carers, they found that the skill regime enacts conflicting strategies—lowering the barriers for Singaporean citizens to enter the elderly care industry while raising standards for migrant domestic workers. Ozkan (2018)’s cross-national comparison of foreign qualification recognition processes for pharmacists also indicates that migrants’ capacity for mobility is not only determined by their own human capital but also shaped by the local systems of professional regulation and immigrant selection.

To examine the context-dependent construction of “skills” in migration, Liu-Farrer et al. (2021) proposed an analytical framework with the following inquiries: *Who* are the arbiters of skill? *What* constitutes skill? *How* is skill constructed in the migration process, and in turn, how does skill affect mobility? These questions urge researchers to identify the concrete agents and mechanisms in the local formation of migrant skill regimes, moving the questions from the macro structure to the meso organizations.

Building on the previous literature, I argue that we not only need to examine the constitution of migration carework skills at the intersection of care, migration, and skill regimes on the macro level, but should also identify the “skilling” and “deskilling” processes on the meso and micro levels, in which migrant workers interact with different gatekeepers across their migration journey. I also challenge the assumption of a linear relationship between skill, desirability, and labor market mobility. Cross-national comparison is a fruitful research design that highlights the local institutional and cultural contexts that define and measure what constitutes migrant and carework skills, leading to different migration pathways and labor market consequences for individual workers.

I identify three major gatekeepers in the migrant carework skill regime: the receiving state, the sending state, and the intermediary. The receiving state plays the role of skill arbiter through a multitude of policy measures concerning migrant care workers, including visa categorization (skilled or unskilled migrants), certification and training requirements (prerequisite skills and on-the-job training), and labor market allocation (duration of contract, rights to job transfer, possibility of promotion, etc.). These policies are often outcomes of political negotiation, involving a variety of organized interest groups representing employers, brokers, professional organizations, and local workers (Oishi, 2021).

The sending government is another critical gatekeeper in the skill regime of care migration; they may restrict or promote the emigration of health workers (Cabanda, 2017). Many sending states have built up a training and certification system for prospective migrants that helps professionalize their transnational workforce and secure the nation’s competitive edge in the global labor market (Chang, 2018; Guevarra, 2010; Ortiga, 2017). These programs also offer an educational infrastructure of “pastoral empowerment” in which aspiring migrants are not only expected to internalize self-discipline as a “good worker” but also cultivate the skills of self-advocacy when a violation of labor rights takes place (Parrenas, 2021).

Finally, the intermediary agents are essential links in the migrant skill regime, serving as arms and legs that materialize policy regulations from both the receiving and the sending end. They provide critical migration infrastructure that facilitates but also conditions migrant workers’ mobilities (Xiang & Lindquist, 2014). Axelsson et al. (2022) have urged us to break down the state-intermediary divide and view their relationship as “co-production of the regulatory space of labor migration.” For instance, the sending states usually outsource pre-departure training and orientation to recruitment agencies or nongovernment organizations (Guevarra, 2010). Many receiving governments, such as Taiwan, Hong Kong, and Singapore, shift responsibilities for immigration control to brokers or employers (Tseng & Wang, 2013). States may also delegate training and management to nonprofit and civic actors, such as South Korea’s EPS (Employment Permit System) program (Surak, 2018).

This article focuses on the receiving end of migrant care skill regimes, including receiving state governance in cooperation with the intermediary agents. More specifically, I ask the following three questions: How do Japan and Taiwan define *what* constitutes appropriate skills for migrant caregivers and *who* can be suitably equipped to become qualified workers and future citizens? How do the receiving states govern the process of recruitment by incorporating other intermediary agents, including commercial brokers and nonprofit organizations? How do the above policies and regulations affect migrant care workers’ mobilities?

### Why compare Taiwan and Japan?

The cases of Japan and Taiwan are comparable due to similarities in their demographic structures and welfare systems. They both have a rapidly aging population coupled with a declining birthrate. Until recently, both countries have maintained strict immigration policies, including a framework of ethnicized citizenship primarily based on the descent principle and rigid regulations concerning the naturalization of foreigners. Unlike South Korea, which recruits co-ethnic (Korean Chinese) as the primary source of care workers, Japan and Taiwan do not have the option to incorporate diaspora labor in the care sector and rely on migrants from Southeast Asia (Ogawa, 2018).

Despite the above similarities, the two countries have adopted divergent ways to recruit migrant care workers. Taiwan officially started a “guest worker” program in 1989 to recruit “unskilled foreigners” and deprive them of access to long-term residency which is nevertheless available to professional foreigners. The total number of migrant workers exceeded 710,000 at the beginning of 2020; one-third of them were hired to care for the elderly, ill, or disabled. Migrant caregivers are mostly hired by private households; less than 6% work in medical or care facilities. Indonesia is the major country of origin (76%), followed by Vietnam (12%) and the Philippines (11%).^1^

By contrast, Japan has long been perceived as a “negative case” among liberal democracies due to its restrictive policies for unskilled foreign labor (Bartram, 2000; Komine, 2018). Recently, Japan started to implement several schemes to accept migrant care workers, but the number is very limited compared to its aging population. There are no official statistics on the number of foreign care workers, but the estimated total is about 3500, mostly from the Philippines and Indonesia (Kakuta, 2017, as cited in Carlos & Suzuki, 2020). Another notable difference from Taiwan is that all migrant caregivers in Japan are placed in care facilities and not allowed to work in private homes, reflecting the care regime of institutional professionalism (Lan, 2018).

The recent developments in these two countries demonstrate paradoxical contrasts worthy of empirical investigation and theoretical exploration. Despite being categorized as “unskilled” workers, migrant caregivers in Taiwan have gained increasing labor market mobility. In contrast, although most migrant caregivers in Japan have nursing backgrounds or degrees in their country of origin, they encounter difficulty in pursuing labor market mobility due to their cultural and linguistic differences.

This comparative study draws on a broad range of government documents, policy reports, journalistic coverage, secondary literature, and interviews with experts, including government officials, scholars, and activists. I also conducted interviews with 15 recruitment agency staffers in Taiwan during 2020–2021. In Japan (Tokyo and Osaka areas), I interviewed eight recruiters, two care work and language instructors, and four care facility managers in 2019.

### Migrant care workers in Taiwan: “unskilled” but desirable

In 1992, Taiwan opened the gate for the employment of “foreign domestic helpers” for households with children under the age of 12, and “foreign caregivers” for the elderly and sick. In contrast to the strict regulation on the employment of foreign domestic helpers, it is much easier to hire a caregiver for the elderly or the sick.^2^ Migrant care workers are expected to provide custodial care and standby service on a live-in basis, playing the role of a surrogate family member to help adult children fulfill the filial duty of taking care of their aging parents (Lan, 2006). The demand for live-in elderly care is so rampant that the qualification for employment was further relaxed in 2016 to cover seniors with mild dementia or above 85 years old.

Taiwan has implemented two ten-year National Long-Term Care (LTC) plans since 2007; the current program provides seniors with some hours of home care, meal programs, and community daycare centers. However, the eligibility of seniors is based on a means test and the availability of family support, and the coverage remains very limited (Chien, 2018; Wang & Chan, 2017). Despite being named “social welfare foreign workers” by the government, migrant care workers are not included in the LTC scheme. In addition, migrant caregivers employed by private households are not protected by the Labor Standards Law and are thus not eligible for the protections of Taiwan’s minimum wage, overtime pay, and off-day requirements. The average monthly wage of migrant care workers now is within the range of NT$17,000–22,000.^3^ They are entitled to one day off each week, but many only have one day off per month (compensated with overtime pay for NT$560 per day) or even less.

Migrant live-in caregivers are expected to provide flexible, round-the-clock service to meet the needs or convenience of their employers. Their job duties vary across households: some perform nursing care, such as assisting with eating, toileting, or bathing, for frail or bedridden patients, while others are mostly responsible for domestic work such as cooking and cleaning. Their less visible duty or skill is that of emotional labor, which is intensive for those who care for elders with dementia and/or who need to cope with multiple members of the employer’s household (Yu, 2015).

The market demand for migrant workers has pressured the government to extend the duration of their residency. The maximum duration of a work permit for migrant workers was initially only 3 years, but was gradually extended to six, nine, and, now, 12 years for factory workers and 14 years for caregivers. However, they are still deprived of the rights to permanent settlement or naturalization.

### *Privatizing governance *via* commercial brokers*

The privatization of recruitment and training characterizes the labor migration regime in Taiwan. Tseng and Wang (2013) have described its model of state governance as “governing at a distance”: the government outsources to commercial brokers and employers the management of migrant workers, especially concerning their whereabouts and departures at the end of their contracts.

Although the government stipulates that foreign care workers must receive a minimum of 90 h of training before they come to Taiwan, there is little regulation of the curriculum. The training courses, designed by overseas recruitment agencies, usually cover basic knowledge and skills for housework, babysitting, elder care, cooking Chinese food, and how to use modern electronic appliances such as vacuum cleaners and microwaves. The instructors are usually returned migrants with no professional nursing backgrounds. The limited medical knowledge and nursing skills with which migrants have equipped mismatches with the “overstretched responsibility” they assume as caregivers in Taiwan; many are asked to perform acts of medical intervention for patients, such as suction of sputum or tube feeding (Ogawa, 2020).

A substantial amount of time is spent on learning Mandarin Chinese, especially in preparation for the migrants’ forthcoming interviews with brokers from Taiwan. The language instruction and drills are designed to affirm workers’ docility to employers’ commands (Chang, 2018) and cultivate an embodied aptitude of “deferential competence” (Lan, 2016). Although basic Mandarin skills are essential for migrant caregivers to communicate with employers and clients, language fluency is not always preferred because it may intrude on family privacy or increase migrant workers’ tendency to “run away” (Lan, 2006).

In Indonesia, an aspiring migrant worker stays in the training center for 2 months, on average, before her departure overseas. The training camp serves other functions in addition to skill transfer. The trainees are usually not allowed to leave the center and must follow routine schedules, wear uniforms, and live a collective life. All these measures aim to produce docile bodies through everyday discipline (Liang, 2011). Moreover, due to the increasing difficulty of recruiting a sufficient number of migrant care workers, the agency now offers an aspiring migrant (or her family) a small settlement fee (approximately US$170) upon checking in, and the migrant remains confined to the center until being assigned to an overseas job. In other words, the training camp serves as a labor storehouse for the agency, while creating a relationship of indebtedness between the migrant and her recruitment agency (Killias, 2018).

The changing practice of placement fee collection also indicates the increasing desirability of migrant workers in the regional labor market. In the late 1990s and 2000s, a prospective migrant care worker had to pay between NT$90,000 to NT$160,000 for a job placement in Taiwan. Most of the recruitment costs were borne by the migrant workers themselves, while employers paid little for the brokerage service (Lan, 2006). The situation has nevertheless changed by the mid-2000s. Nowadays, to hire a care worker, the Taiwanese agency first collects a fee from the employer (NT$15,000–30,000), and uses the money to pay overseas brokers to secure workers’ résumés for interviews and selection. A Taiwanese agency that I interviewed described the situation as follows:

The caregiver market has now become a seller’s market. Taiwanese agencies now have to “buy” the résumés (of migrant workers) from an overseas agency. I have to pay before importing labor; otherwise, I have no résumés to show to my clients (employers in Taiwan). You pay NT$15,000 to 20,000, and then they will give you probably one or two résumés.

Before departing for Taiwan, migrant care workers today pay nothing but instead receive a settlement fee (NT$5000) from Indonesian brokers; the loan payment is also substantially decreased (NT$6000 per month for 6 months or less). In other words, employers and brokers have to pay in advance to “buy a worker” (Chang, 2021). By contrast, the higher-paying positions of factory work are more attractive and thus migrants are willing to “buy a job” by paying a fee as much as USD$6000.

The decline of placement fees for migrant care workers occurred as a combined result of market forces and interventions by the sending states. During the 1990s and 2000s, migrant labor demand, then under strict quota control, was significantly lower than overseas labor supply. Taiwan’s brokers and employers thus held greater bargaining power in their transactions with recruitment agencies from sending countries. Nowadays, with new job opportunities in higher-salary receiving countries, including Japan and South Korea, migrant workers do not always prefer Taiwan as their top destination, especially for care work that requires living with the employer.

The sending states have also established a system of training and credentials to increase the value of migrant labor. The Indonesian government mandates that first-time prospective domestic and care workers must undergo 200–600 h of training for information and preparation (Chang, 2021). Colleges and universities in the Philippines also offer a wide array of postsecondary degrees for both nurses and caregivers (Ortiga, 2017). The shifting market dynamics allow the sending states to bargain with receiving governments and negotiate better terms for their overseas nationals. The Indonesian government has stipulated that the recruitment costs for migrant workers, including air tickets, passports, medical examinations, etc., be less than about 50,000 Taiwan dollars. In 2020, it further proposed the goal of “zero recruitment fee,” but the policy has not yet been implemented under the impact of the Covid-19 pandemic.

#### Labor market consequence: movements across sectors

In Taiwan, the categorization of “unskilled” has deprived migrant workers of access to long-term settlement so they can only navigate limited mobility across visa categories or destination countries. Some migrant caregivers have married their employers or other Taiwanese citizens, transferring to the track of marriage migration (Lan, 2006). Some continue to emigrate as caregivers but pursue “stepwise migration” by moving to higher-ranked destination countries (Paul, 2017).

More recently, migrant workers gained some access to labor market mobility after Taiwan’s government relaxed the regulations on employer transfer and visa duration. In 2008, the Ministry of Labour (MOL) allowed migrant workers to switch employers before the contract expires if both employers and workers agree to terminate the contract. Since 2016, migrant workers have been allowed to stay in Taiwan and switch to new employers at the end of a contract, without being forced to leave Taiwan and pay the placement fees again to start a new contract. These policy changes happened largely due to pressure from the humanitarian infrastructure (Xiang & Lindquist, 2014), including the international community, especially the US State Department’s “Trafficking in Persons Report,” and the advocacy of local non-government organizations.

The pandemic further opened some space for migrant care workers to pursue labor market mobility. The strict border controls related to Covid-19 have disrupted the entry both of newly hired migrant workers and of those who returned home for vacation. The labor shortage is even more serious in the semiconductor and related industries, which are willing to hire migrants who transfer from care work despite their lack of relevant work experience or skills. The media have reported on the increase in migrant shortages and migrant job transfers under sensational headlines such as “The Flight of Foreign Maids” (TVBS, 2021).^4^ Migrant care workers in residence have also gained increased bargaining power vis-à-vis their employers and brokers during the pandemic. Some have asked for a raise, while others have threatened to “run away” if not allowed to transfer to less demanding care work or factory jobs (Lan, 2022).

Facing rising pressure from household employers, the government tried to constrain migrant workers’ mobilities during and after the peak of the pandemic. Taiwan imposed a nationwide three-month Covid-19 Level 3 Alert from May 19 to July 26, 2021.^5^ A few cluster infections broke out at migrant dormitories in Miaoli County. On June 6, the MOL placed a temporary ban on practically all transfers of migrant workers across employers.^6^ On August 28, the MOL announced new rules that limit migrant workers' ability to transfer to new jobs across different sectors.^7^ In 2022, after a series of negotiation with the Indonesian government, the MOL raised the minimum wage of migrant caregivers from NT$17,000 to NT$20,000 for those who sign contracts after August 10 to increase their incentive to stay in care work.

The increasing labor shortage and international competition for foreign labor have pressured Taiwan to restructure the binary hierarchy of “skilled/unskilled” migration regime. On the one hand, by launching a new scheme of an Employment Golden Card in February 2018 and passing the Act for the Recruitment and Employment of Foreign Professionals in July 2021, the migration regime has become increasingly flexible to facilitate the mobility of global talent by providing tax benefits and widened access to permanent residency.

On the other hand, the government has recently set up a pathway for blue-collar migrants to pursue long-term residency. Starting from April 30, 2022, the MOL announced the “Retention of Foreign Intermediate Skilled Workforce Program.”^8^ Migrant workers can shift to a new visa status called “mid-level technical worker” if certain requirements are met, including working in Taiwan for more than 6 years, earning a monthly salary above the designated amounts,^9^ and holding some language and professional skills. Care workers, for instance, need to pass the Mandarin Language Proficiency Test Basic Level A2 and complete a 20-h online education training course. After holding this visa for more than 5 years, they will become eligible to apply for permanent residency. In the new policy, the measurement of skills has become more malleable, shifting from credentials to ability, experience, and wage level, but the threshold is still high for migrant caregivers because care work is undervalued and underpaid.

### Migrant care workers in Japan: skilled and culturally inadequate

Japan’s Long-Term Care Insurance (LTCI) was implemented in 2000 to provide universalized care for the elderly. The rapid expansion of social care requires a growing pool of qualified care workers. Despite the significant labor shortage in this sector, Japan has slowly opened the door to foreign care workers. The first program to accept migrant care workers was based on Economic Partnership Agreements (EPAs) with Indonesia (effective in May 2008), the Philippines (October 2008), and Vietnam (October 2009). EPA “care worker candidates” (*kaigo fukushishi*) acquire nursing training before entering the program—they need to graduate from a nursing college or vocational school, or obtain a caregiver certificate accredited by their home government. In Japan, they can only work for medical institutions or care facilities. Although LTCI subsidizes “home helpers” who visit their clients a few hours per day, foreign caregivers are not allowed to take on this task.

The EPA is positioned as an on-job-training program. The candidates receive a four-year visa for “specific activities,” and they can continue employment and residency in Japan after passing the national exams to become formally registered nurses or certified care workers. Despite the substantial costs invested in the EPA program, the size of recruitment is very limited, estimated to be 4300 persons in total (Asato, 2019). The number of foreign applicants has been on the decline in recent years, sometimes attracting even fewer applicants than the number of slots offered by long-term care facilities (Carlos & Suzuki, 2020). The program is therefore criticized for being “designed to fail” (Vogt, 2018).

In 2017, the Japanese government revised the Immigration Law to launch some new schemes for the recruitment of migrant care workers. First, it added the category of care work to the TITP (Technical Intern Training Program) program, which started in 1993 with the alleged goal of transferring skills to developing countries but which has become a “side door” for labor migration (Oishi, 2005). Starting in November 2017, a trainee can work as a caregiver at nursing homes in Japan for 3 years, with the possibility of extending for another 2 years. Before they arrive in Japan, they need to acquire some on-the-job experience in care work, and prior to applying they must pass the N4 test of Japanese-language proficiency, which includes basic Japanese daily conversation.

Second, the 2017 reform also created a new residence status, “*kaigo*” (carework), for foreign students who graduate from the two-year vocational course offered by care worker training institutions and receive care worker certification. The duration of the *kaigo* visa is a minimum of one year and a maximum of 5 years, without limitation on the number of renewals, and *kaigo* visa holders are allowed to bring their family to Japan. This new scheme has encouraged Japanese language schools and nursing homes to recruit more care work overseas students (*kaigoryugakusei*) (Ogawa, 2020). A student is allowed to work for a maximum of 28 h per week during the school term and eight hours a day during school holidays, usually in the sponsoring long-term care facilities. As an interim provision, students who graduated by March 2022 automatically receive a certified care worker license, even without passing the licensure examination, as long as they work in a long-term care facility for five consecutive years (Carlos & Suzuki, 2020).

Finally, the Abe government launched the “Specified Skilled Worker (SSW) Visa No. 1” program in April 2019, which opens up employment for migrant workers in 14 sectors, including nursing care, for up to a total of 5 years. The government expected up to receive 60,000 workers in the nursing care business over the following 5 years from 2019. Foreign workers in this program are positioned as “mid-level skilled” but their skill levels are equivalent to those of the TITP. The SSW visa holders are given more generous entitlement than unskilled workers, including transferring employers freely and the possibility for permanent residency, provided they can pass the certification exam and shift to the *kaigo* visa.

Why did Japan implement such a complicated system, involving multiple tracks of care migration? When I posed the question to Japanese officials, a common explanation was that these different tracks of care migration serve different purposes: the EPA program is to facilitate free trade; TITP helps to transfer skills to less developed countries; and SSW aims to recruit semi-skilled migrant workers. These multiple tracks are under the jurisdiction of distinct administrative offices: EPA under the Ministry of Economics, TITP under the Ministry of Labor, and SSP under the Ministry of Justice.

Japan’s multi-track system is especially puzzling when contrasted with South Korea, which had a similar trainee system but abolished it completely by replacing it with a government-managed guest worker scheme (EPS) in 2003 (Kim, 2020). Instead, Japan has maintained and even expanded the TITP program to incorporate migrant care workers. According to Milly (2017), business elites dominated the early policy forum and set the agenda for using TITP. The Japan Association of Certified Care workers opposed the use of TITP and insisted on the certificate, so the proposal was later modified by specifying the requirement of language skills and establishing professional pathways. This policy direction also aligns with the general social perception in Japan. In a 2010 government survey, Japanese respondents considered the most important qualifications for foreign workers to be “Japanese language skills,” “understanding Japanese customs,” and “understanding Japanese culture,” while “professional skills and knowledge” was considered of lesser priority (The Cabinet Office, 2010).

The addition of SSW indicates continuity and also discontinuity with the existing migration regime. Because Japanese politicians have resisted the import of “simple labor,” the SSW program maintains the façade of skilled migration. However, the definition of “skilled migrants” was significantly changed in the 2018 reform, shifting “from a credential-based to an ability-based concept” (Oishi, 2021). It officially recognizes the “skills” of SSW workers based on prior knowledge, techniques, and work experience, instead of academic degrees or professional certifications.

#### *Paternalistic governance *via* supervisory organizations*

The TITP program relies on the intermediary of 組合(カケル) or “supervising organizations,” which connect recruitment agencies in the sending countries (“sending organization”), employers (“implementing organizations”), and the Japanese bureaucracy. Although the supervising organizations are supposed to be non-profit organizations such as the chamber of commerce, in reality, they are not that different from for-profit placement agencies. They work with the sending organizations to recruit potential migrants and match them with employers via virtual or on-site interviews. Care worker trainees usually receive pre-departure training for 3 months and, upon arrival in Japan, stay in a dormitory–training center under the supervision of receiving organizations for another month. During their stay in Japan, at least one staff member from the supervising organization is assigned as the everyday life advisor for the new migrants. Yoshida (2020) describes this as a style of “paternalistic labor management”—the supervisory organizations are mandated to monitor both the trainees’ working situation and their private lives, especially to prevent them from running away.

Throughout the process, the supervisory organizations have to prepare and submit a plethora of documents, including skill evaluations, language exam certificates, training plans, and life plans, because the TITP trainees are supposed to be learning skills rather than working in Japan. The supervisory organization staff members told me that they need to prepare and submit more than 60 documents for a single application. The amount of required paperwork has lessened slightly due to the pandemic, but hard copies of each application are still required. Not unlike Taiwan, the Japanese government also governs at a distance and outsources its paternalistic duties to the supervisory organizations; it further plays the role of “governing by documentation” by monitoring the process of placement, training, and management through cumbersome documentation requirements without direct intervention.

Red-tape bureaucracy and the complex tracks of recruitment create high information costs for migrants and space of rent-seeking for brokers. In their study of foreign students going to Japan, Liu-Farrer and Tran (2019) have argued that the more regulations there are, the more indispensable brokerage becomes to the migration process. Not only do migrants rely on intermediary organizations to navigate the complicated system, but nursing homes also need these intermediaries to facilitate the documentation. In particular, supervising organizations staffed by OB (“old boys”)—a term that generally describes alumni from a particular school or institution, and that here refers to retired government officials—can mobilize their previous working experience and social ties to speed up the applications.

Japan sets higher requirements than Taiwan for the training hours and language skills of migrant caregivers. The EPA candidates are provided with one year of free Japanese-language training and are required to pass the Japanese Language Proficiency Test (JLPT) N3. The language requirement for *kaigo* students is even higher (the JLPT N2). However, trainees usually receive pre-departure training for 6 months in their home country until they pass the JLPT N4, which is the second lowest level and requires very basic conversation. After arriving in Japan, they must stay at a training center run by the supervisory organization for another 40 days before working in a nursing home.

This emphasis on language and cultural knowledge is embedded in the organizational culture of Japanese nursing homes. The celebration of traditions and rituals is a major characteristic of Japanese nursing homes; a sense of cultural intimacy helps the residents feel comfortable with living in an institution (Świtek, 2016; Wu, 2004). The employers expect migrant caregivers to acquire intimate knowledge of Japanese language and culture, especially of the proper use of honorifics (*Keiko*). Migrant caregivers are usually taught only the forms that could be used to converse politely with people who do not belong to one’s group (Świtek, 2016: p. 98). Recently, some activists have advocated speaking “Yasashii Nihongo” (simple/easy Japanese) to facilitate communication with migrant workers (Lee & Niya, 2021), but the language form is criticized for sounding childish and failing to deliver respect to the Japanese elderly.

The training regime that emphasizes linguistic skills and cultural knowledge costs time and money. Compared to Taiwanese employers, Japanese employers cover a much larger share of recruitment costs (about US$20,000), including pre- and post-departure training, documents, airfare, and management fees. There are two major reasons behind this. First, even for the recruitment of local workers, Japanese employers are used to paying a significant fee to an intermediary organization such as a dispatching company. Second, facility owners and managers I interviewed reported that they are willing to pay more for “quality labor.” Migrant workers are preferred not only due to their medical backgrounds and nursing skills but because contract-bound foreigners provide more labor force stability. Migrant workers are also more flexible in their working hours and willing to accommodate working overtime and the night shift.

Although Japanese employers cover most recruitment costs, a migrant worker still has to pay an average of US$3600–5000 to the sending agency, according to my interviews with supervisory organization staff members. For those who enter the track of *kaigo* student migration, the training costs for language skills are even higher. One needs to have an N2 language proficiency level or be enrolled for at least 8 months in a Japanese language school in Japan. Language schools and care worker training institutions charge almost the same tuition fees—about US$7200–7700 per year (Carlos & Suzuki, 2020). In addition, some of the employer costs may be transferred to migrant workers surreptitiously, creating a relationship of debt-bondage (Ogawa, 2021).

#### Labor market consequence: (Im)mobility across the visa hierarchy

Despite the language and cultural distance, migrant workers are popular in Japanese care facilities, according to the interviews of owners by Ogawa (2020). They are appreciated for their cheerful personalities and soft skills in the conduct of emotional work, although such capacities are often assumed to be a cultural habit rather than a hard-earned skill (Lan, 2018). In addition, migrant workers’ limited Japanese-language skills do restrict their ability to take on more responsibility or gain promotions in Japanese facilities because care workers are expected to write care plans for individual clients and communicate formally with their families (Ogawa, 2020).

The labor market mobility of TITP workers is further constrained. The contract period is strictly set for a maximum of 5 years. Trainees are not allowed to change employers or take a side job. Transfers are allowed only when they are fired for reasons that concern the company they work for, such as bankruptcy. International *kaigo* students are also subject to constrained mobility. If they receive a scholarship or a tuition subsidy from a local government or care facility, they are obliged to work in that region or facility for a few years after graduation and sometimes face deductions from their future wages.

Compared to other tracks of care work migration, SSW offers more possibilities for mobility and settlement. Although SSW migrants are categorized as “skilled migrants,” the skill requirements, including passing a vocational exam and N4 Japanese, are not much higher than the requirements of TITP. Like highly skilled foreigners, SSW workers are entitled to employer transfer and permanent residency. Direct hiring is possible, although manpower agencies and dispatching companies can register as “support organizations” for SSW deployment. Japan has ratified the ILO (International Labor Organization) 181 Convention and endorsed the zero-recruitment-fee policy, and the SSW program is touted for its successful abolishment of commercial agencies to prevent exploitative practices (Oishi, 2021). However, Asato (2019) has criticized the laissez-faire governance of the SSW program, which views job-seeking as an individual pursuit and places no regulations on the intermediary infrastructure.

In practice, both employers and brokers prefer TITP to SSW precisely because of the latter’s potential for labor market mobility. Only employers or brokers who had failed to extend the contract for a TITP trainee or who were trying to re-recruit a TITP returnee would use the channel of SSW. An interviewed supervising organization staffer, a naturalized Japanese citizen originally from China, boldly criticized the contradictory nature of the SSW program, which showcases protection for migrant rights and yet perpetuates the exploitative system of TITP. He said:

The Japanese government must have planned this from the beginning. They simply want to extend TITP by adding another 5 years with SSW.… The program is full of contradiction, only asking for Japanese N4 and nothing else.… They do this for Americans, who have been criticizing TITP. This is for the Olympics.… It’s all about surface and reality. Japan has been maintaining the surface. It is a guest worker program by nature, but they just deny it.

As elsewhere, the pandemic and related measures of border closure have disrupted the recruitment of migrant workers in Japan. According to my follow-up interview with one supervising organization staffer, an increasing number of TITP trainees transferred their visa status to SSW during the pandemic. In November 2021, the Ministry of Justice announced an expansion of the scope of permanent residency for blue-collar migrant workers in 2022 (Japan Times, 2021), although no concrete plans have been released so far.

Despite the institutional possibility of staying in Japan permanently, the linguistic and cultural barriers to doing so remain substantial for migrant care workers to overcome. It is challenging for Filipino and Indonesian migrant caregivers to study while working intensively within a short-term contract. Even for the EPA candidates with more protective hours of study, the passing rate has been low due to the high threshold of language proficiency; among those who passed, many left Japan to either return to their home country or work in another destination (Carlos & Suzuki, 2020).

Based on my interviews with Chinese brokers and facility owners, it is easier for trainees from China to learn Japanese, given the similarity between Japanese kanji and Chinese characters, and to pass the language tests and certification exams. A professional certificate helps migrants not only prolong employment and residency but also gain recognition and respect from Japanese coworkers and clients. In fact, Japanese citizens do not need a license to work in nursing homes, and the wage difference between those with it and those without it is modest.^10^ For a migrant worker, however, a professional certificate is a status shield that helps counterbalance the disadvantage of being a cultural outsider. As one nursing homeowner said, the client would think that “she’s a foreigner but she has a license.”

## Discussion and conclusion

Comparting the paradoxical conditions of migrant care workers in Taiwan and Japan, I propose a multifaced analytical framework to identify the four dimensions of migrant carework skill regimes: migrant skill as a political language and structure of governance, care work skill as social and cultural constructions, the infrastructure of recruitment and training, and the consequence of labor market mobility. Table 1 summarizes the comparison:

**Table 1:** Migrant care skill regimes: comparing Taiwan and Japan

	Taiwan	Japan (TITP)
Paradox	“Unskilled” yet desirable	“Skilled” yet culturally inadequate
Migrant skills as political language and structure of governance	Binary hierarchy of skilled/unskilled	Undesirable “simple labor” Paternalistic “skill transfer”
Care skills as social/cultural construction	Flexible labor and personal care	Professional inclusion and cultural exclusion
Recruitment and training infrastructure	For-profit brokerage Training center as labor storehouse	Supervising organizations Training documentation as bureaucratic governance
Labor market mobility	Horizontal across sectors	Vertical across visa categories

In Taiwan, migrant care workers, who are classified as “unskilled” workers and undesirable citizens, have become very desirable in the regional and local labor market. The migration regime has sustained a binary hierarchy of “skilled/unskilled” division until the recent policy changes. The political language of “unskilled” justifies the deprivation of rights and citizenship from guest workers despite their essential labor contribution. The familialistic care regime, which recruits live-in caregivers as filial surrogates, has slowed the development of Long-Term Care plans and increased the desirability of migrant care workers, especially during the pandemic. Meanwhile, the state “governs at a distance” by outsourcing most recruitment and training to commercial brokers. The pre-departure training centers deliver limited knowledge and skills to intended migrants but serve as labor storehouses. Instead, migrant care workers acquire nursing and emotional skills to accommodate the demand for flexible labor and personal care in individual households. The recent shortage of migrant labor supply and deregulation of employer transfer has opened up space for experienced workers to bargain for better terms or pursue *horizontal* movement across sectors.

Japan’s migrant care skill regime is a mixture of *professional inclusion* and *cultural exclusion*. Migrant care workers in Japan are entitled to *vertical* mobility across different visa categories, but this opportunity is restricted in reality. The Japanese government chose TITP program, a thinly veiled guest worker program, to expand the recruitment of migrant caregivers and continues to frame it as the benevolent mission of “skill transfer” for less-developed sending countries. The state governance operates through cumbersome documentation on job applications and training plans, thus delegating to the supervising organizations the paternalistic duty of monitoring migrant workers’ work and personal lives. Migrant caregivers are allowed to work in institutional settings only and must go through a lengthy process of skill training. The facilities expect them to acquire sufficient language skills and cultural knowledge, and constrain their responsibilities and mobility for the same reason.

This comparative study offers important theoretical implications for migration researchers. The multifaceted analytic framework uncovers the context-dependent constitution of “skill” at the intersection of migration, care and skill regimes. Cross-national comparisons further untangle the relationship between skill, desirability, and labor market mobility. I also demonstrate that the intermediary infrastructure of training and certification goes beyond skill transfer; it also serves the purposes of migrant labor power storage, bureaucratic governance, and paternalistic control. I urge researchers to further examine multiple institutional and cultural contexts in both sending and receiving countries that enable and condition migrant workers’ capacity to acquire, transfer, and apply particular sets of skills, and to identify the “skilling” and “deskilling” processes and consequences across the different stages of their migration careers.

This study also holds critical policy implications. The severe labor shortage during the pandemic has pressured many receiving countries, including Japan and Taiwan, to relax their regulations on migrant workers’ settlement. It is still too early to assess the implementation and effects of these new measures, but these policies demonstrate that the existing labor migration regimes in both countries are unsustainable. It is important to reconsider both the definition and the evaluation of the skills required for migrant workers. Both sending and receiving states should coordinate with non-state actors to provide skill training and life support, not only to empower migrant workers but also to improve the quality of care they can provide. We should view care work as involving culture-sensitive and emotional-intelligence skills without falling into the trap of cultural essentialism or ethnic otherization. We should also recognize migration as a skill-building process (Oishi, 2021) and offer pathways of mobility and settlement for those who have contributed greatly to their host society.

## Data Availability

Not applicable.

## Abbreviations

EPA: Economic Partnership Agreements (Japan)
EPS: Employment Permit System (South Korea)
ILO: International Labor Organization
JICWEL: Japanese International Corporation of Welfare Service
JLPT: Japanese Language Proficiency Test
LTCI: Long-Term Care Insurance (Japan)
MOL: Ministry of Labour (Taiwan)
SSW: Specified Skilled Worker Visa (Japan)
TITP: Technical Intern Training Program (Japan)

## Footnotes

1 The number of migrant workers in Taiwan declined after March 2020 due to border control measures related to the Covid-19 pandemic. By January 2022, there were a total of 664,733 migrant workers in Taiwan, of whom 225,074 were care workers. Ministry of Labor, Republic of China (Taiwan), https://statdb.mol.gov.tw/evta/jspProxy.aspx?sys=100%26kind=10%26type=1%26funid=wqrymenu2%26cparm1=wq14%26rdm=I4y9dcIi (accessed March 11, 2022).

2 In 2000, the government adopted the Barthel index, which relies on doctors to measure the frailness of the wards as eligibility for hiring a live-in migrant caregiver. Nevertheless, the process of medical assessments for care needs is often manipulated by brokers (Liang 2015).

3 The recent currency exchange rate between USD and NT is in the range of USD1 to NT28–30.

4 In fact, the number who transferred successfully across sectors was still limited, even though the growth rate was substantial. From January 2021 to the end of August 2021, before the MOL placed a restriction on cross-sector job transfers, a total of 2404 caregivers had left their jobs in favor of factory work, compared to only 66 people in all of 2019 and 287 people in all of 2020 (Lan 2022).

5 During this period, people were required to wear masks at all times when leaving homes; indoor gatherings were limited to five people, and ten for outdoor gatherings. The schools were shut down; working from home was encouraged but not required.

6 Exceptions include migrant workers who are victims of violence, sexual harassment, sexual assault, or human trafficking.

7 A migrant care worker has to register for a transfer at a government-run employment service center, which will advertise her service to household employers for 14 days. Only if no employers express interest in hiring her can she start to seek job opportunities in other industries. Because there is a significant shortage of caregivers, it is practically impossible for a migrant care worker to transfer to a factory job today.

8 Foreign Labor Rights Protection Information Website, https://fw.wda.gov.tw/wda-employer/home/mid-foreign-labor/index?locale=en (accessed on August 10, 2022).

9 The monthly salary standards are NT$33,000 for factory workers, NT$29,000 for facility care workers, and NT$24,000 for live-in care workers.

10 The monthly wage of a migrant care worker in Japan is approximately ¥150,000–210,000 and the license premium is about ¥3000 per day. To become eligible to take the national exam for Japanese care worker certification, one needs to acquire 1850 h of schooling or three years of work experience (Ogawa 2020).
